# Neurobiological Determinants of Tobacco Smoking in Schizophrenia

**DOI:** 10.3389/fpsyt.2018.00672

**Published:** 2018-12-06

**Authors:** Aliya M. Lucatch, Darby J. E. Lowe, Rachel C. Clark, Karolina Kozak, Tony P. George

**Affiliations:** ^1^Addictions Division, Centre for Addiction and Mental Health (CAMH), Toronto, ON, Canada; ^2^Institute of Medical Sciences, University of Toronto, Toronto, ON, Canada; ^3^Division and Brain and Therapeutics, Department of Psychiatry, University of Toronto, Toronto, ON, Canada

**Keywords:** schizophrenia, nicotine, tobacco, neurobiology, nicotinic acetylcholine receptor

## Abstract

**Purpose of review:** To provide an overview of the underlying neurobiology of tobacco smoking in schizophrenia, and implications for treatment of this comorbidity.

**Recent findings:** Explanations for heavy tobacco smoking in schizophrenia include pro-cognitive effects of nicotine, and remediation of the underlying pathophysiology of schizophrenia. Nicotine may ameliorate neurochemical deficits through nicotine acetylcholine receptors (nAChRs) located on the dopamine, glutamate, and GABA neurons. Neurophysiological indices including electroencephalography, electromyography, and smooth pursuit eye movement (SPEM) paradigms may be biomarkers for underlying neuronal imbalances that contribute to the specific risk of tobacco smoking initiation, maintenance, and difficulty quitting within schizophrenia. Moreover, several social factors including socioeconomic factors and permissive smoking culture in mental health facilities, may contribute to the smoking behaviors (initiation, maintenance, and inability to quit smoking) within this disorder.

**Summary:** Tobacco smoking may alleviate specific symptoms associated with schizophrenia. Understanding the neurobiological underpinnings and psychosocial determinants of this comorbidity may better explain these potential beneficial effects, while also providing important insights into effective treatments for smoking cessation.

## Introduction

The high rates of tobacco use in the schizophrenia (SZ) population are widely recognized, but the underlying neurobiological factors contributing to this comorbidity are not fully understood. Rates of tobacco smoking are between 45 and 88% in SZ compared to <16% of the general population (1, 2). In this review, we aim to highlight the recent literature on the latter category of neurobiological determinants and discuss some potential treatment targets.

The high prevalence of smoking in SZ is maintained in large part by resistance to quitting (3); quit rates from an American nationally representative sample range from 10 to 27.2% for those with psychotic disorders compared to 42.5% in the general population (4). Additionally, relapse rates pose a common challenge in delivering cessation treatments, but there is some indication that longer courses of pharmacological treatments could reduce the possibility of relapse (2). Unfortunately, these high smoking rates come with a cost, and smokers with SZ are at higher risk for tobacco-related morbidity and mortality; people with SZ have ~25 years of shortened lifespan, with 53% of this being related to tobacco-smoking conditions (5–7). One population-based study in the United States (U.S.) found that among individuals with SZ, cardiovascular disease, lung cancer, and respiratory diseases such as chronic obstructive pulmonary disease and pneumonia contributed to the most deaths (8). It is clear from the evidence that reducing smoking rates has the potential to drastically change mortality rates and improve outcomes for these patients.

## Etiology of Tobacco Smoking in SZ

Many explanations have been proposed for the higher prevalence of smoking in persons with SZ. In this section, we briefly review some of these important factors before highlighting current findings on the neurobiology of this comorbidity. These major factors include increased craving in SZ, modulating negative symptoms, pro-cognitive effects of nicotine, and genetic factors (3). In addition, we compare the self-medication hypothesis with the addiction vulnerability hypothesis for tobacco use in SZ.

It has been proposed that due to the pathophysiology of SZ, these individuals may have an enhanced experience of the reinforcing effects of nicotine (3, 9). This idea has been corroborated with a study that compared smokers with SZ to non-psychiatric control smokers in an abstinence condition; they found that the SZ group reported stronger cravings and withdrawal symptoms and had a shorter time to smoking lapse compared to the control group (10). This effect was moderated by negative affect and withdrawal symptom severity (10). Another study using an animal model produced lesions in the ventral hippocampus, a region associated with SZ, and found increased reinforcing effects and drug-seeking behavior for nicotine (11).

There appears to be a link between the enhanced reinforcing effects of nicotine and the role that negative affect has on increasing the smoking rate in SZ. This may be due to the deficits in reward processing and alterations in reward-related brain circuitry that is characteristic of negative symptoms in SZ (12, 13). In an fMRI study of smokers with SZ compared to control smokers, researchers found that both groups had brain reactivity to smoking cues, but SZ group had reduced brain reactivity to neutral cues, and that this effect was related to negative symptoms (14). This finding indicates that the enhanced addictive properties of nicotine in SZ is not due to a stronger reactivity to nicotine-related cues, but rather may be related to the underlying negative symptomatology (14). A recent study using a cognitive assessment of reward learning in smokers found a negative correlation between general reward responsiveness and intensity of nicotine craving (15). This finding suggests that individuals with negative affect and a dysfunctional reward circuit, such as those with SZ, may be more susceptible to nicotine addiction (15). Together, these findings indicate that increased negative symptomatology may play a role in the enhanced susceptibility to smoking in SZ, yet does not reveal the full picture.

### Pro-cognitive Effects of Nicotine in SZ

Another key etiological factor to consider is the potential pro-cognitive effects that nicotine has on SZ; nonetheless there have been mixed findings in this field. For instance, much of the epidemiological research surrounding this comorbidity has found no effect or worsened cognition within chronic smokers with SZ (16–19). However, lack of control for time since last cigarette may result in nicotine withdrawal-related cognitive impairment (20–22) and may explain some of these negative findings as the participants were likely to be experiencing significant smoking deprivation. Table 1 has been included below to illustrate the variety of study methodologies examining the pro-cognitive effects of smoking and how each study accounted for the duration since last cigarette. However, studies that carefully control for time since last cigarette have found that smoking produces cognitive deficits in SZ, particularly in working memory, visual learning, and attention (3, 23, 24, 31). In laboratory studies where nicotine is acutely administered or acute overnight abstinence and reinstatement paradigms are used (thereby avoiding any confounding effects of nicotine withdrawal), smoking groups have shown marked improvement for attention, working memory, pre-pulse inhibition, visuospatial working memory, processing speed, and verbal learning and memory(23, 24, 26, 28–37).

**Table 1:** Cognitive Effects in Acute vs. Chronic Smokers with SZ.

**Study**	**Study design**	**Control for time since last cigarette**	**Findings**
(16)	Cross-sectional	No control for last cigarette	= cognition No significant differences in cognitive outcomes between smokers and non-smokers with first-episode SZ
(17)	Cross-sectional	Last cigarette an hour prior to testing	↓ cognition Treatment-resistant SZ smokers performed worse on problem-solving cognitive domain compared to smokers. Other cognitive domains were not different between the groups.
(18)	Cross-sectional	No control for last cigarette	↓ cognition Current smokers with SZ or bipolar disorder had worse composite cognitive function compared to non-smokers.
(19)	Cross-sectional	No control for last cigarette	= cognition No significant differences in cognitive outcomes between smokers and non-smokers with first-episode SZ
(23)	Prospective human laboratory study	Deprived of cigarettes for 2 h and given either nicotine or placebo-containing gum	↓ cognition Attention was significantly improved in non-smokers compared to smokers with SZ after nicotine administration.
(24)	Cross-sectional	Last cigarette an hour before testing, cognition administered 2 h in, allowed smoke breaks with 30 min interval before re-initiating cognitive testing	↓ cognition Visual learning significantly improved in non-smokers compared to smokers.
(25)	Cross-sectional	Frequent smoke breaks (smokers never abstinent for >30 min)	↑ cognition Sustained attention, processing speed, response inhibition were significantly improved in smokers compared to non-smokers with SZ. No differences in non-psychiatric controls.
(26)	Cross-sectional	Frequent smoke breaks (smokers never abstinent for >30 min)	↑ cognition Verbal memory was significantly increased in smokers compared to non-smokers with SZ.
(27)	Cross-sectional	No control for last cigarette	↑ cognition Processing speed, spatial working memory, and visual learning was significantly improved in smokers compared to non-smokers with SZ.
(28)	RCT of haloperidol x nicotine	Overnight abstinence with randomized dose of nicotine patches	↑ cognition Nicotine lead to a dose-related reversal of haloperidol-induced cognitive impairments in memory and reaction time.
(29)	Placebo controlled crossover for cigarettes and nicotine nasal spray in current smokers	Administration of nicotine nasal spray or placebo nasal spray, and high nicotine cigarette and denicotinized cigarette.	↑ cognition Nicotine in nasal spray lead to significant improvement on a spatial organization task, verbal memory, and reaction time in SZ. Both cigarettes lead to improvement on spatial organization task.
(30)	Placebo controlled crossover with nicotine and placebo patch	Withdrawn from tobacco and given nicotine patch or placebo patch	↑ cognition Improved performance on n-back (working memory and selective attention) task in SZ smokers vs. non-smokers and worsened performance in control smokers vs. non-smokers
(31)	Cross sectional–3 conditions	3 test conditions—baseline, overnight abstinence, and 1 h after reinstatement with no more than 15 min smoking deprivation	↑ cognition Impaired visuospatial working memory (VSWM) during overnight abstinence in SZ, improved VSWM and CPT upon reinstatement in SZ.
(32)	Cross sectional–3 conditions	3 test conditions—baseline, overnight abstinence, and 3 h nicotine patch	↑ cognition Reaction time was significantly increased in the nicotine patch condition and worse in the abstinence condition in SZ.
(33)	Cross sectional–2 conditions	2 test conditions—after overnight abstinence, normal smoking behavior (No control for last cigarette)	↑ cognition VSWM was significantly increased in the smokers with SZ compared to healthy controls
(34)	Cross sectional	No control for last cigarette	↑ cognition Divided attention was significantly increased in the smoking condition and worse in the abstinence condition in SZ.

Studies have also compared cognitive performance between non-smoking and smoking patients with SZ. These studies account for the level of nicotine in the participant's system by allowing frequent smoke breaks so as to avoid inducing a state of withdrawal (25, 26). Non-smokers revealed significantly worse cognitive deficits, particularly in verbal memory (25, 26).

Interestingly, this effect is specific to those with schizophrenia, as no such finding was observed in patients with major depression, bipolar disorder or non-psychiatric controls (25, 26). Furthermore, individuals categorized as ultra-high-risk (UHR) for developing psychosis may also demonstrate this effect (27).

Other studies employed cognitive testing in both satiated and abstinent states and demonstrated that smokers with SZ show improvements in various cognitive domains (28–30, 33, 38). These studies are shown in Table 1. A satiated state was produced by administering nicotine throughout the study session with a patch, gum, or nasal spray, providing the benefit of acute nicotine exposure (28–30, 32); however, these were regular smokers. There are few studies examining acute nicotine administration in non-smokers, due to the nature of tobacco use disorder and the all-or-nothing tendency for people to be regular smokers or non-smokers. The few studies that have examined nicotine administration in non-smokers found an overall improvement in attention following nicotine administration and a specific effect at improving cognitive outcomes in the SZ participants (23, 39). Nonetheless, nicotine administration improves cognitive outcomes in SZ individuals, this may account for the increased frequency and severity of tobacco use in SZ and also the perseverance of tobacco use disorder in this population (40).

There are two primary theories proposed to explain the pro-cognitive effects of nicotine in SZ, and the relationship to the increase prevalence of smoking in SZ. First, the self-medication hypothesis proposes that individuals with SZ choose to smoke to alleviate the clinical symptoms and cognitive deficits that are characteristic of the illness as well as the side effects of antipsychotic medications (41). Many of the studies examining the procognitive effects of nicotine lend support to the theory, but others have refuted this theory. For example, one group found that it was a stronger tendency for those with SZ to experience withdrawal when abstinent that led to cognitive deficits and that blood nicotine concentration did not affect performance when compared to healthy controls (42). In response to these challenges identified with the self-medication hypothesis, researchers have developed an alternate theory to explain the heightened prevalence of smoking in SZ which is termed the addiction vulnerability hypothesis (43). This theory proposes that it is due to genetic, neurobiological, and environmental factors that are inherent to the SZ diagnosis that make these patients susceptible to smoking (43). Understanding the unique factors contributing this vulnerability can provide us with novel treatments targeting smoking cessation in this specific population, in particular, building our knowledge about the underlying neurotransmitter systems and brain circuitry is essential (44).

### Neurobiological Determinants of Tobacco Smoking in Schizophrenia

#### Nicotine

Nicotine, the addictive component in tobacco cigarettes, binds to nicotinic acetylcholine receptors (nAChRs), which are endogenously expressed in the human brain and influenced by the native agonist, acetylcholine (45). nAChRs are a heterogeneous family of ion channels, that are expressed on various cellular regions of both excitatory and inhibitory neurons, allowing for modulation of neurotransmitters (45, 46). The composition of the nAChRs is a variety of subunits which define the receptors' actions and properties (47). The most common high-affinity nAChRs include receptors consisting of two α4 subunits, two β2 subunits, and an undefined fifth subunit (48). Single nucleotide polymorphisms (SNPs) found on the CHRNA4 gene coding for the α4 subunit has been associated with nicotine dependence (49–51). Another important nAChR type to consider is that of the α7 receptor, which consists entirely of α7 subunits. SNPs located on the receptor coding gene CHRNA7, has been associated with both SZ diagnoses (52, 53) and nicotine dependence (54). Notably, there are two nicotinic acetylcholine receptors (nAChR) subtypes linked to cognition: high-affinity α4β2 and low-affinity α7 nAChRs (45, 55). High-affinity nAChRs are sensitive to nicotinic antagonists such as mecamylamine (56), and mediate nicotine reinforcement and cognition (36, 57), whereas low-affinity nAChRs are less nicotine-sensitive.

Dopamine, norepinephrine, serotonin, glutamate, aminobutyric acid (GABA) and opioid peptides are neurotransmitter systems influenced by nAChRs (58). The neurobiological phenotype of SZ involves dysfunction of similar neurotransmitters, such as the dopaminergic, glutamatergic and GABAergic systems, as well as overarching dysfunction of cortical and subcortical communicative circuitry. Developmental and genetically predisposed abnormalities observed in the prefrontal and hippocampal regions in individuals with SZ may facilitate neural circuitry dysfunction, promoting a vulnerability toward addiction, such as tobacco use disorder (59). The neurobiological abnormalities of SZ will be discussed after which the corresponding effects of nicotine will be supplemented in order to provide insight into this prevalent comorbidity.

### Pathophysiology of SZ and Nicotine Effects

**Figure 1:**
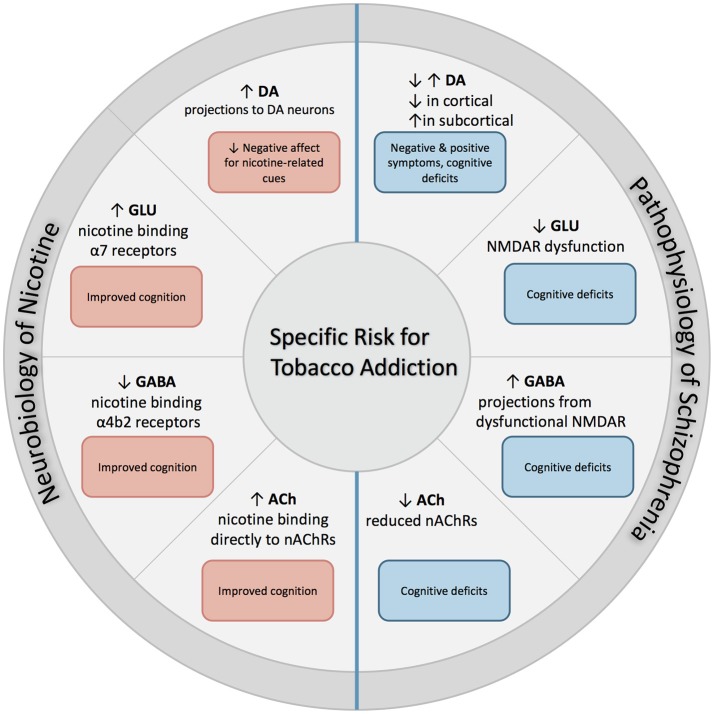
Excessive dopaminergic activity has been proposed within subcortical regions in individuals with SZ, and is associated with positive symptoms of SZ. Conversely, a hypo-dopaminergic state has been postulated in the cortical regions, and is associated with cognitive deficits and increased negative symptoms. NMDAR abnormalities found in SZ contribute to both hypo-glutamatergic activity and hyper-GABAergic activity, and leads to cognitive dysfunction. Individuals with SZ have reduced expression of nAChRs which leads to altered nicotinic cholinergic transmission, which may contribute to cognitive dysfunction. When nicotine is administered through tobacco smoking, these deficits may be partially attenuated. First, nicotine binds directly to nAChRs that are located in mesolimbic dopaminergic pathways, which increases its expression and contributes to reduction in negative affect in response to smoking-related cues. In addition, nicotine binds to α7 and α4β2 receptors on glutamatergic and GABAergic neurons in the prefrontal cortex, attenuating deficits found in SZ and enhancing cognition.

The following section describes the role of each neurotransmitter system on both nicotine addiction and schizophrenia. An overall summary and simplification of these effects is illustrated with Figure 1.

#### Dopaminergic Dysfunction

The dopamine (DA) hypothesis for SZ features the imbalances in dopaminergic neurotransmission throughout the brain, such as presynaptic abnormalities of DA neurons that are described in both SZ and high-risk populations (60, 61). DA dysfunction influences both cortical and subcortical circuitry, facilitating symptomatology of SZ differentially. Subcortical regions in the brain have been associated with increased dopaminergic activity, leading to over-stimulation of D2 receptors (62, 63). Hyper-dopaminergic activity in subcortical regions, specifically in the associative striatum, has been associated with positive symptoms of SZ, including psychosis (61, 64–66). Cortical regions, however, have been linked with dampened dopaminergic activity. This has been investigated through various functional imaging studies, demonstrating an under-stimulation of D1 receptors in the frontal regions of individuals with SZ (67). Additionally, *in vivo* findings of decreased dopaminergic activity in the dorsolateral prefrontal cortex has been associated with cognitive impairment severity, such as worsened working memory, as well as negative symptom severity (68–71). Treatment that targets dopamine dysfunction, the most pervasive form of medication for SZ (targeting dopamine D2 receptors), has proven to aid with positive symptoms; however, in individuals who lack substantial dopaminergic dysfunction, this treatment does not robustly align with symptom improvement (61, 72).

#### Glutamatergic and GABAergic Dysfunction

A more recent hypothesis for SZ pathology involves glutamatergic dysfunction, involving the hypofunction of *N*-methyl-D-aspartate (NMDA) receptors of which glutamate, the major excitatory transmitter in the brain, binds (73, 74). Supported by genetic convergence, brain tissue analysis and brain imaging studies (74–76), the glutamatergic theory offers a unique conceptualization that encompasses the widespread deficits observed in SZ (73). For example, NMDAR antagonists, such as phencyclidine and ketamine, correlate with the emergence of both negative and positive symptoms as well as general neuropsychological and sensory deficits associated with SZ in contrast to amphetamine, a dopamine receptor agonist, which is mostly associated with inducing the positive symptoms of the disorder (73). NMDA receptors influence the majority of input, output and interneuronal cortical projections and, therefore, have a diffuse influence on brain function (77). In SZ, glutamatergic dysfunction due to NMDAR abnormalities has been noted in regions within the limbic system, the hippocampus and the dorsolateral prefrontal cortex (78–81). NMDAR activity is also important in considering the functioning and maintenance of the brain's main inhibitory transmitter -aminobutyric acid (GABA) (82). Deficits in GABA synthesis (deficits in glutamic acid decarboxylase (GAD)-67, which aids GABA synthesis) have been denoted throughout the cortex of individuals with SZ (83). Decreased functioning of GABAergic interneurons has been posed to contribute to cognitive impairment in SZ by means of decreased synchronization in neuronal cortical activity (84). Specifically, the hypofunctionality of NMDARs in SZ has been proposed to lead to dysfunctional GABAergic transmission (85). Dampened activity of NMDARs at GABAergic interneurons disrupts and reduces inhibitory control over cortical activity as well as the overall synchrony of gamma oscillations, leading to clinical disruptions in SZ (86).

Additionally, NMDARs, which influence both glutamate and GABA, highly influence dopamine synthesis and transmission (75, 87). Glutamate, stimulated by NMDARs, regulates dopamine neurons that project from the ventral tegmentum area (VTA) toward the nucleus accumbens (NAc) or the prefrontal cortex (PFC), as well as GABA neurons that also play a role in regulating dopamine neurons (88). Dysfunction within the glutamatergic system in SZ has been proposed to facilitate the dopaminergic dysfunction linked to cognitive disruptions of the illness (87, 89). Additionally, presynaptic dopamine released both subcortically and within the frontal regions of the brain are influenced by inhibitory GABA interneurons, therefore disruption in GABA signaling via NMDAR abnormalities has also been linked to abnormalities in dopamine signaling (73, 90, 91).

#### DA, GLU, GABA, and Nicotine

Nicotine modulates dopaminergic transmission in both subcortical and cortical regions. Primary dopaminergic projections involve transmission from the VTA toward the NAc; major components of reward circuitry in addiction of which nicotine enhances through this pathway (92, 93). Nicotine is able to influence dopamine's activity by directly binding nAChRs on dopaminergic projections sourced in the VTA as well as by regulating glutamate and GABA activity, which excites and inhibits dopaminergic activity, respectively (94). Specifically, nicotine binds to the α7 receptors along glutamatergic neurons, stimulating their activity and enhancing NMDAR function, which together enhances dopaminergic neuronal activity (94). Nicotine also binds α4β2, a high-affinity receptor along GABA neurons, which, with chronic nicotine use, become desensitized, therefore dampening the inhibition on dopaminergic transmission from the VTA to the NAc, while α7, low-affinity nAChRs on glutamatergic neurons are less prone to desensitization, therefore continue to enhance dopamine transmission (94, 95). Nicotine directly binds nAChRs along dopamine neuronal cells, facilitating burst firing and increased dopamine activity directly (96), which, combined with enhanced glutamatergic tone, leads to an overall increased level of dopamine transmission and release in the NAc that supports the reinforcing effects of nicotine (97).

It has been posed that nicotine leads to increases in dopamine levels within prefrontal regions through direct and indirect (GABA and glutamate influences) enhancement of dopaminergic activity that makes up for the lowered dopamine D1 stimulation and ensuing cognitive deficits observed in SZ (44, 67, 98). Nicotine facilitates increased dopamine in the cortex similarly to observations in subcortical regions in that the drug binds high-affinity nAChRs on dopamine neurons that project from the VTA, but in this case toward the cortex, and binds low-affinity receptors on excitatory glutamatergic neurons projecting toward the prefrontal cortex (99, 100). The reported beneficial effects that nicotine influences in the frontal cortex have been proposed to be largely due to its effects on the α7 nAChR subunit, although there is some evidence surrounding α4b2 receptor subunit activity leading to improved higher cognition (98). Because GABA contains many α7 as well as α4b2 nAChRs, nicotine could counteract the deficits observed in GABAergic transmission in SZ and the coinciding prefrontal dysfunction by stabilizing cortical inhibition through enhancing interneuronal activity and frontal gamma oscillations (3, 83, 101–103).

#### naChRs and Nicotine

Additionally, SZ involves the dysregulation of both high- and low-affinity nAChRs (98, 104). Studies have shown that individuals with SZ have a reduction in nAChRs expression throughout brain regions that are central to higher cognitive functioning (105). Additionally, it has been found that chronic nicotine use leads to nAChR receptor desensitization and inactivation during stages of withdrawal, which are reactivated upon overnight abstinence (3, 106, 107). Clinically, this pattern of receptor desensitization may explain the phenomenon where smokers prefer the first cigarette in the morning, and why cognitive deficits are present during periods of withdrawal (106). However, in the SZ population, this pattern of desensitization and resensitization may have a different presentation due to the decreased expression of nicotinic receptors, which may account for their increased severity of tobacco addiction (108).

SZ has also been linked genetically to the CHRNA7 gene, a potential site of genetic heritability, which codes for the α7 subunit of nAChRs (109). Individuals with SZ who smoke have exhibited improvements in their cognition, highlighting the potential benefits of stimulating this receptor in this population (26, 29). For example, nAChR agonists and antagonists, such as varenicline and mecamylamine, have been used in various smoking cessation and treatment trials in which the results further support the cognitive improvements observed in smokers with SZ (31, 36, 110). Additionally, levels of CHRNA7 protein and mRNA became comparable to non-psychiatric smokers following smoking in SZ (53). Overall, nAChR dysfunction may influence the aberrant signaling of glutamate, GABA, and dopamine of which nicotine use may partially alleviate (3).

#### Circuitry Dysfunction

It is posed that each transmitter system, including dopamine, glutamate, GABA and cholinergic neuronal transmission, incorporates a circuit, supported by genetic risk, that facilitates a risk for SZ presentation (61, 111). The dysfunction within SZ and nicotine's influence on these abnormalities do not exist in isolation. The combination of abnormal dopamine neurotransmission and nAChR signaling, along with imbalances in glutamate and GABA transmission, which influences the former dysfunctions, may lead to the widespread deficits and symptoms observed in SZ (3). Nicotine stimulates nAChRs, which are situated along glutamatergic and GABAergic neurons. Nicotine, therefore, may modulate glutamate-GABA interactions and normalize excitation-inhibition influences over dopamine signaling and communication within the brain through improving baseline nAChR-stimulation dysregulated in SZ. The influence nicotine has on the transmission and general circuitry in SZ has been shown to alleviate certain symptomatic characteristics and cognitive deficits, as described above, which may place this population at an enhanced risk to developing tobacco use disorder.

#### Tobacco Use and Antipsychotic Medication

Cigarette smoking has been found to increase activity of the liver enzyme, cytochrome P450 1A2 (CYP 1A2), which in turn break down drugs in the body, including antipsychotic medications such as olanzapine and clozapine (112). As a result, there is reduced concentration of these antipsychotics in medicated SZ smokers, which predictably leads to a reduction in side effects, motivating further use (113) (supporting the self-medication hypothesis). An important implication of the reduction in antipsychotic medication is the potential for worsened symptoms of psychosis (114), to account for this, researchers of a meta-analysis study have indicated that smokers with SZ should be prescribed antipsychotics at a dose double that of non-smokers (115).

### Biomarkers of Vulnerability

#### P50 Suppression and Mismatch Negativity

P50 suppression is an electroencephalographic measure of cortical inhibition that follows a second tone that is presented 500 ms after an initial tone (116). SZ, as well as the pathological and heritable characteristics of the disorder, is associated with the sensory gating deficit of diminished suppression (117–119). This deficit has been genetically linked to polymorphisms found on the promoter region of the CHRNA7 coding gene for the α7 nAChR subunit, as well as decreased function of the α7 nAChR (120–122). It is thought that GABAergic neurotransmission within the hippocampal region, which is dysfunctional in SZ, mediates the production of P50 suppression, therefore may contribute to this population's sensory deficit (123). Nicotine, through its influence on nAChRs and the downstream effects, has been shown to remediate the deficits in P50 suppression for this population (35, 124, 125). Moreover, nAChR agonists also show improvements in cognitive functioning, including P50 suppression (126).

MMN is a neurophysiological test that quantitatively and temporally measures central auditory functioning or, more specifically, the neuronal processing in response to an auditory “oddball paradigm,” which involves the intervention of a deviant stimulus within sequential auditory tones (127, 128). From clinically high-risk to chronic classifications of SZ, this population exhibits reductions in MMN amplitudes based on frontocentral electroencephalographic recordings that are shown across all dimensions of auditory deviance (127–130). This deficit has been linked to NMDAR dysfunction, which correlates to the glutamatergic hypothesis of SZ pathology (119, 131, 132). Nicotine seems to enhance the duration of MMN amplitude, facilitating improvement in this neurophysiological deficit (119, 133, 134).

#### Pre-pulse Inhibition (PPI)

PPI is an electromyography measure of eye blink responses (i.e., one's eye muscle movement) to a startling auditory tone. If a “prepulse” tone occurs before the main auditory stimulus, one's blinking response is attenuated; however, individuals with SZ exhibit a deficit in this gating response (135, 136). This deficit has been associated with CHRNA3 polymorphisms, relating to nAChR dysfunction that is characteristic of SZ, and observed as heritable within this population (137–140). Nicotine has been noted to improve this deficit in smokers with and without SZ (137, 141, 142). Furthermore, abstinence related deficits in SZ (vs. non-psychiatric controls) were ameliorated by smoking reinstatement and blocked by nAChR antagonist, mecamylamine (143), suggesting that nAChR stimulation may remediate PPI deficits in SZ.

#### Smooth Pursuit Eye Movement

Smooth pursuit eye movement (SPEM) tasks involve the measurement of saccades, which are eye movements toward a target stimulus, as well as anti-saccades, which involves the movement away from a stimulus. Individuals with SZ have more intruding saccades to the extent of being described as a heritable characteristic of the diagnosis (144–147). Nicotine has shown to influence this measurement by improving the reliability of saccadic measures in individuals with SZ, but not in non-psychiatric controls, by potentially lowering the hyperactivation in regions facilitating this response and improving cortical inhibitory control (148–152).

## Psychosocial Determinants of Tobacco Addiction in Schizophrenia

There are a variety of psychosocial factors that increase the vulnerability of individuals with SZ to develop and sustain tobacco addictions. Individuals with SZ tend to be of lower socioeconomic status compared to general population, which is associated with an increased likelihood of smoking initiation and a decreased likelihood of smoking cessation (153, 154). These individuals often have fixed, government-assisted income and may spend close to 30% of this income on cigarettes (155).

Elements of the mental health system itself may make individuals afflicted with SZ more vulnerable to smoking. There is a longstanding and pervasive smoking culture in mental health institutions that tolerates and even encourages tobacco consumption (156). Although, the smoking culture impacts all people with mental illness, SZ patients are particularly likely to be exposed, as they receive treatment largely in institutions and mental health settings (5). In fact, 80% of light smokers and 57% of moderate smokers have actually been found to increase their cigarette consumption following psychiatric admission (157). Despite the fact that programs to treat tobacco addiction in inpatient settings have been shown to be effective, mental health staff are reluctant to treat nicotine dependence in psychiatric patients and counseling for smoking cessation is rarely provided (156, 158, 159). Moreover, they are hesitant to ban cigarette smoking in institutions because of concern over patient resistance, infringing on patients' right to smoke and potential negative effects of smoking cessation on treatment outcomes (160, 161). Despite this common concern, inpatient psychiatric facilities that have implemented smoking bans, have demonstrated positive outcomes and had far fewer problems than anticipated (162, 163).

## Treatment Implications

The advantage of broadening our understanding about the underlying neurobiology of this comorbidity is that it may lead to more novel, targeted treatments to be developed. In this section, we will briefly discuss some new developments in the area of treating smokers with SZ. Some major advancements in this field have been drugs targeting nAChRs and the potential for neuromodulation.

Currently, the most commonly studied and accepted treatments for this population in the order of effectiveness have been varenicline, bupropion and nicotine replacement therapy (NRT) (164). Varenicline acts as a partial agonist at the α4β2 nAChR, while buproprion acts at several targets including at the norepinephrine-dopamine receptors and at the nicotinic receptor (165, 166). Pharmacological treatments for smoking are more effective than any behavioral treatments in this population, and maintenance treatment is also an important way to prevent relapse in SZ (164). However, although varenicline has been found effective at reducing overall smoking in SZ, it has not been found to compensate as a cognitive enhancer (167). This is consistent with the findings of a recent meta analysis that determined α7-nAChRs as ineffective treatments for improving cognitive and negative symptom outcomes in SZ based on 8 RCTs (168). Additionally, cessation rates in patients with SZ remain significantly lower than those of the general population, and it is evident that a more holistic treatment strategy is required (164).

Neuromodulation is a promising new treatment modality which may have considerable promise for smokers with SZ (169). For example, repetitive transcranial magnetic stimulation (rTMS) delivers high-frequency magnetic fields to a targeted area of the brain (e.g., dorsolateral prefrontal cortex), stimulating neurons in that region and altering the brain circuitry. One study conducted in our lab found that a short course of rTMS was not effective at reducing craving in an overnight abstinence condition in patients with SZ (170). However, three other studies examined rTMS delivered over a longer course (10, 21, and 28 days, respectively) and found significant improvements on cigarette consumption and craving, but these effects dissipated over time (3, 171, 172). While the mechanism behind the impact of rTMS on nicotine addiction has not be fully elucidated (173), one hypothesis is that rTMS directed to the dorsolateral prefrontal cortex (169) reduces drug craving experienced by the user (174–176) (3. SZ Res.). Another neuromodulation method is transcranial direct current stimulation (tDCS), which provides a weaker electrical current over a longer duration to the brain, modulating neural firing without producing stimulation of neurons (177). A recent RCT of tDCS delivered in 5 sessions over 21 days in smokers with SZ found significant improvements on several cognitive deficits, but no improvements on cigarette use and craving outcomes (178). The findings within the field of neuromodulation are promising, but further studies are needed to corroborate these techniques as an effective treatment for smoking in SZ.

## Conclusions

In summary, it is evident that the comorbidity of SZ and cigarette smoking is widespread, and that the underlying neurobiological factors are complex. Research on these factors is contributing to the development of treatment strategies that may help to reduce smoking and in turn the high mortality rates that arise due to the high smoking prevalence in this population. It is also important to consider a holistic approach because although neurobiology plays a large role in this comorbidity, etiological factors for smoking are multifaceted and all things must be considered. Further research and discussion should continue, and it is important that clinicians work against stigma and toward promoting education about high smoking rates as a specific vulnerability for individuals with SZ.

## Author Contributions

AL: literature review, drafting manuscript, and final submission. DL and RC: literature review and contributing to content. TG: oversight, editor, and finalizing manuscript. KK: literature review for revision recommendations, contributing to content of revisions and final editing.

### Conflict of Interest Statement

The authors declare that the research was conducted in the absence of any commercial or financial relationships that could be construed as a potential conflict of interest.
